# Supplementary material for: Dissecting the precise role of H3K9 methylation in crosstalk with DNA maintenance methylation in mammals
Source: Nat Commun. 2016 Aug 24;7:12464. doi: 10.1038/ncomms12464 (PMC5426519; doi:10.1038/ncomms12464)
Supplement: Supplementary Information — Supplementary Figures 1-5 [file ncomms12464-s1.pdf]

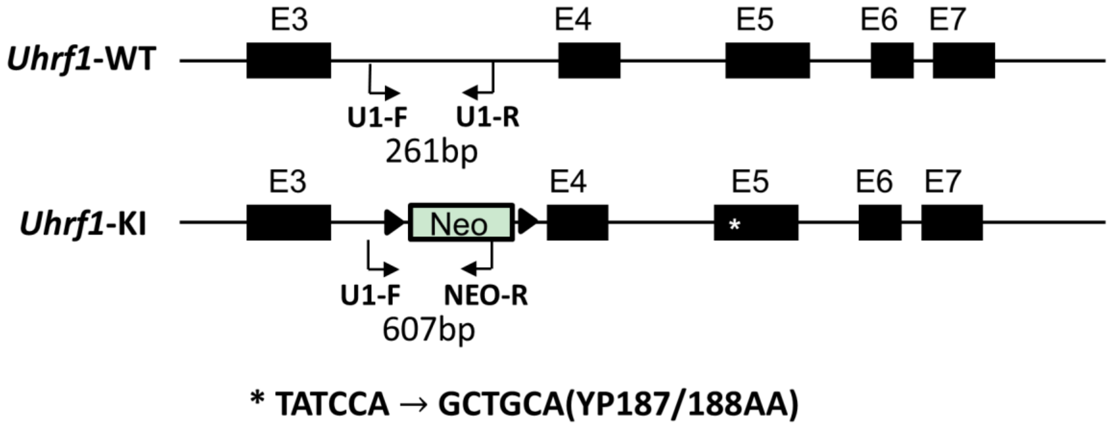

U1-F:GTGGCAGTATAGATTCTCACC
U1-R:CTACCTGGCAGCAAAGC
NEO-R:TATCGCCTTCTTGACGAGTTC

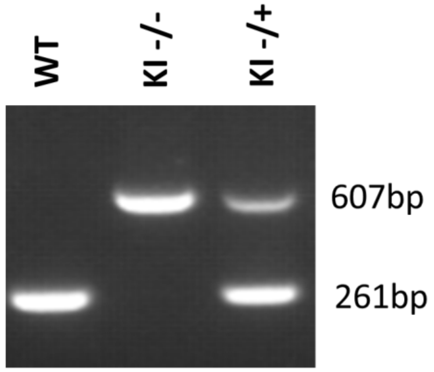

Supplementary Figure 1. Strategy for genotyping of Uhrf1 YP187/188AA KI mice by PCR. The top panel illustrates the genomic difference between WT and KI mice. The middle panel shows the primer sequences for PCR reaction. The lower panel shows a representative genotyping result for WT, homozygous KI -/- and heterozygous KI -/+ mouse.

Figure 1C

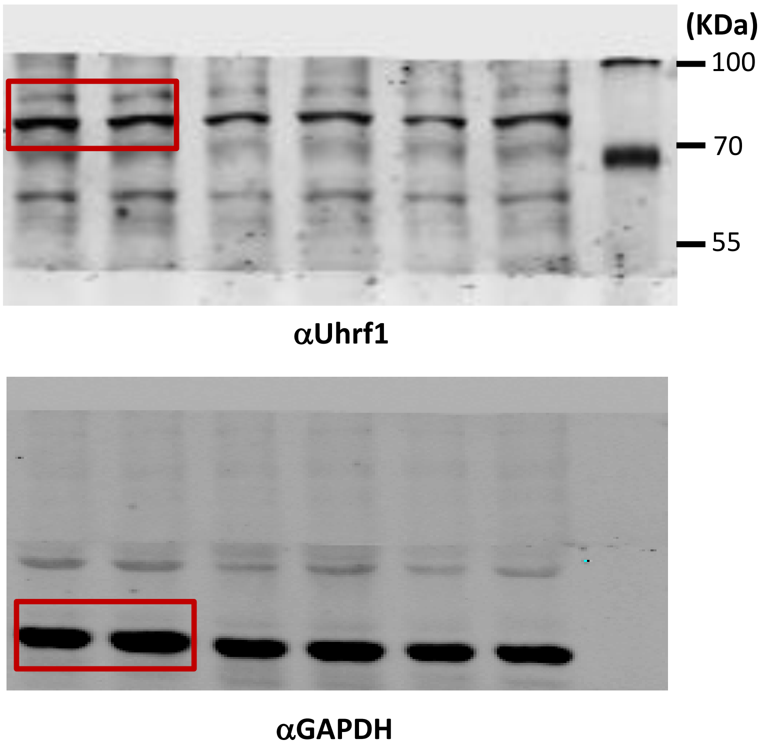

Figure 1D

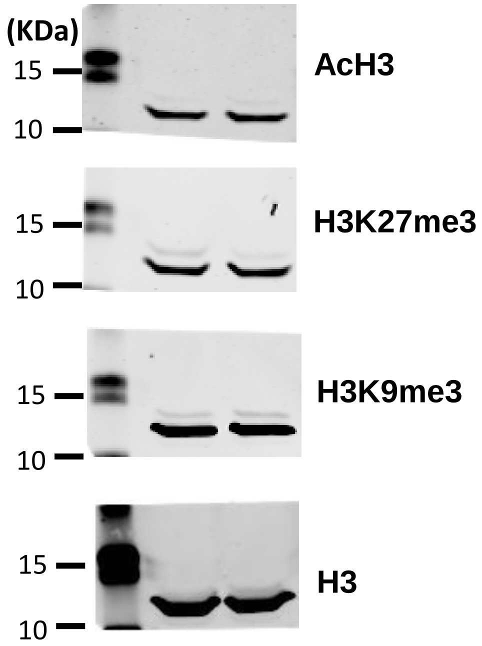

Figure 1E

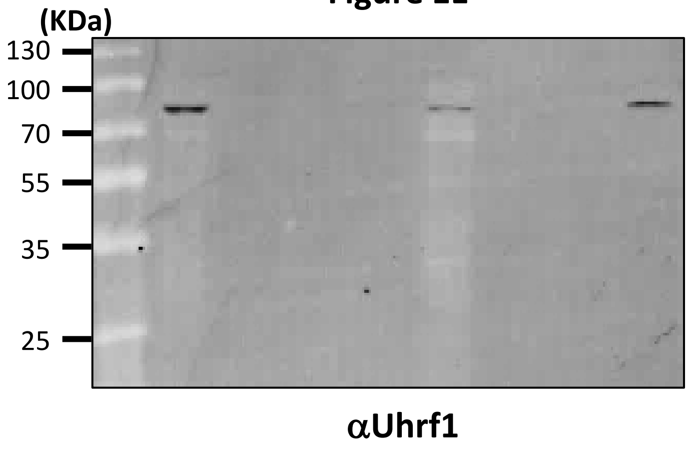

Supplementary Figure 2. Whole uncropped Western blots for Figure 1C, 1D and 1E. Molecular weight markers are as indicated. Labeling and order are consistent with Figure 1.

Figure 3E

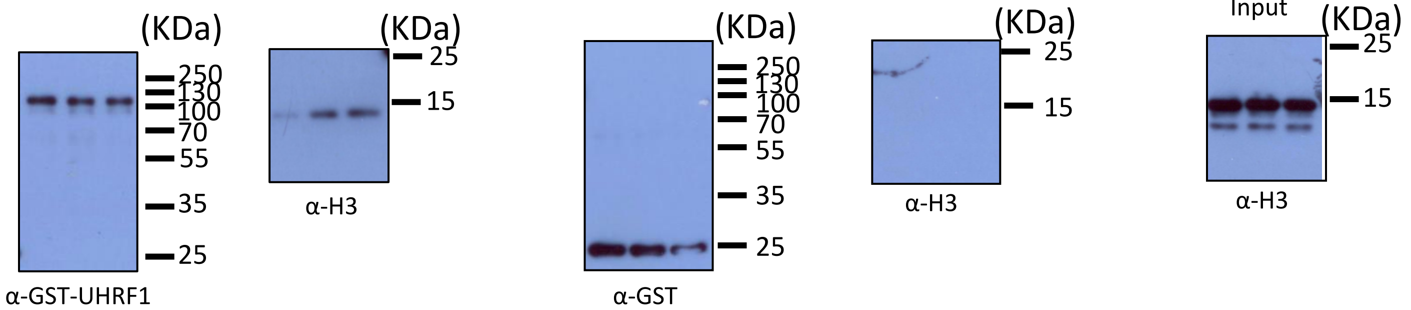

Figure 3G

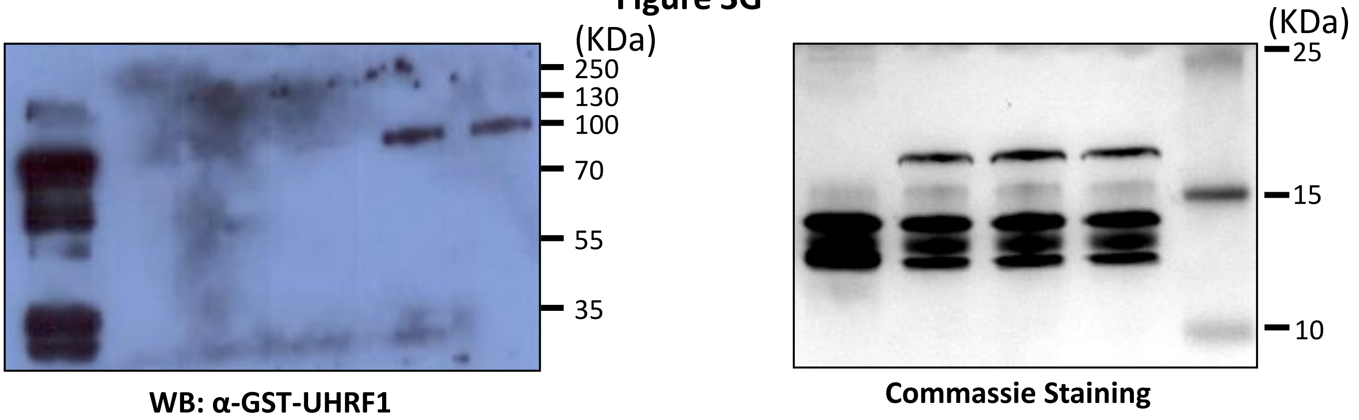

Figure 3H

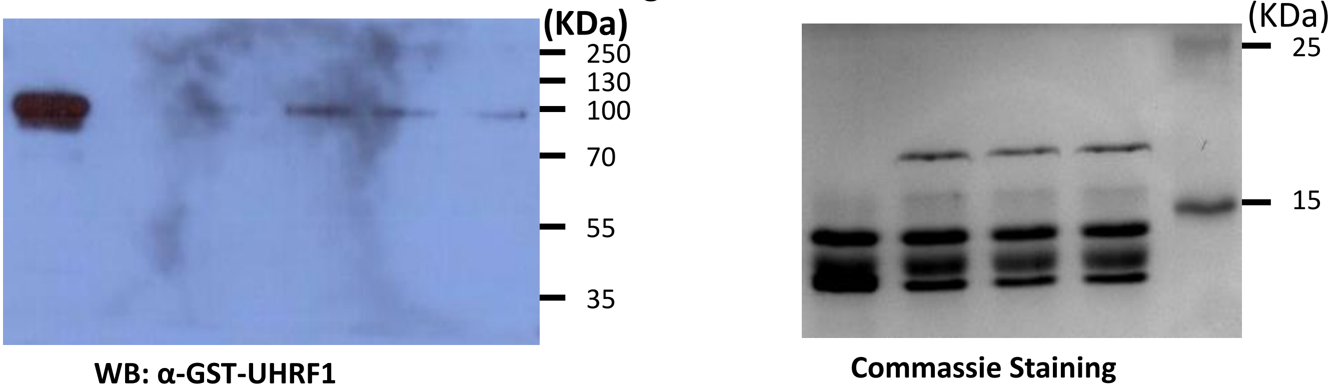

Figure 3I

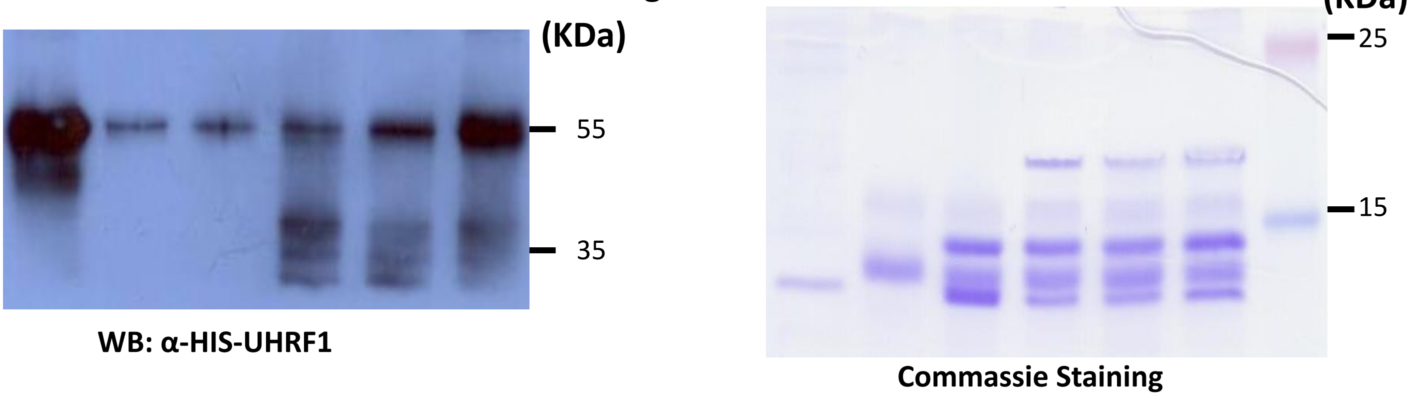

Supplementary Figure 3. Whole uncropped Western blots for Figure 3. Molecular weight markers are as indicated. Labeling and order are consistent with Figure 3.

Figure 4C

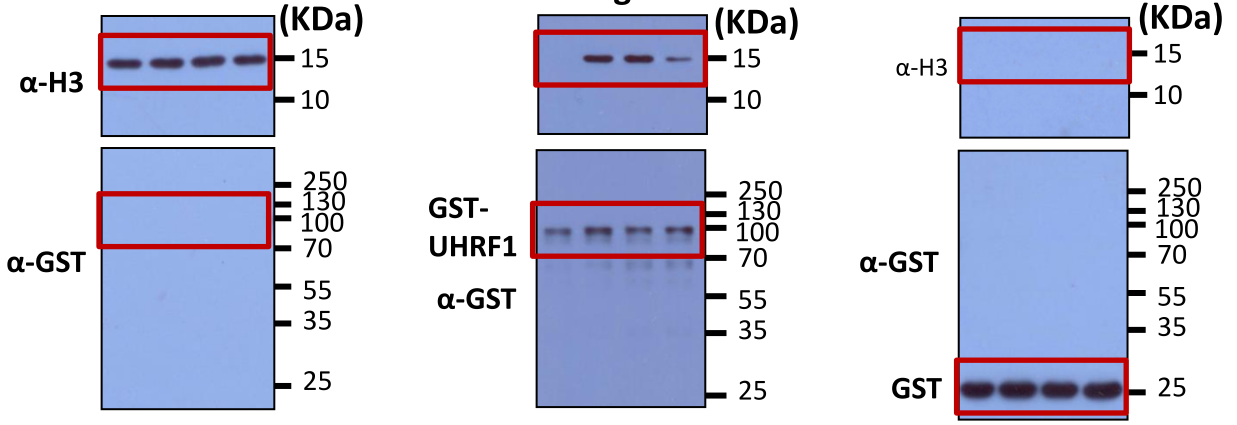

Figure 4D

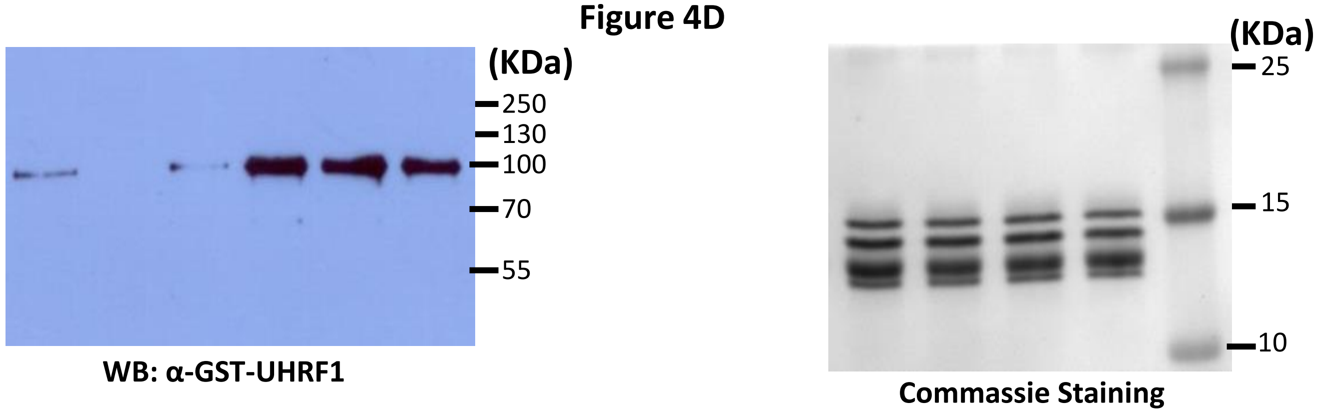

Figure 4E

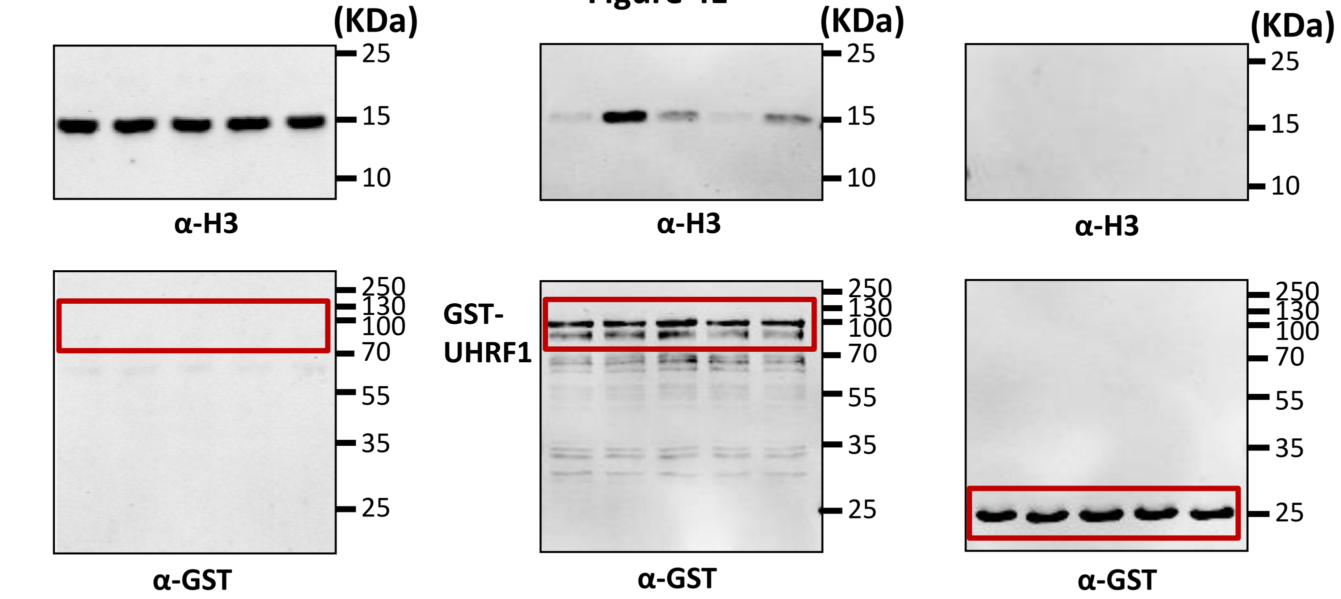

Supplementary Figure 4. Whole uncropped Western blots for Figure 4. Molecular weight markers are as indicated. Labeling and order are consistent with Figure 4.

Figure 5B

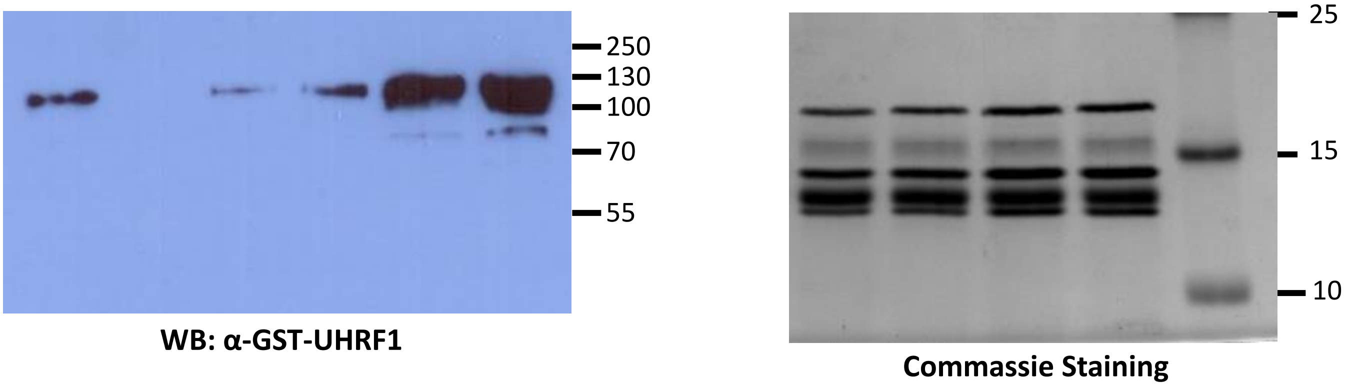

Figure 5D

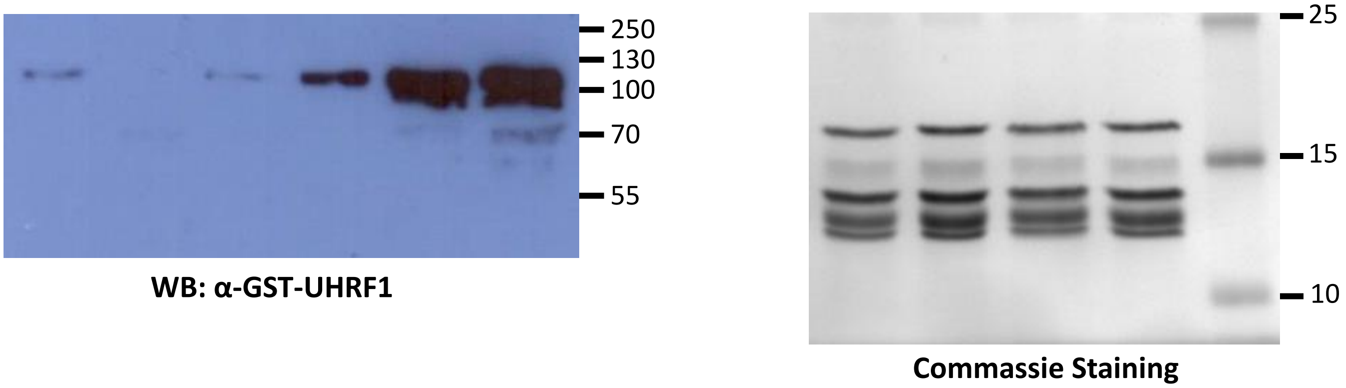

Figure 5F

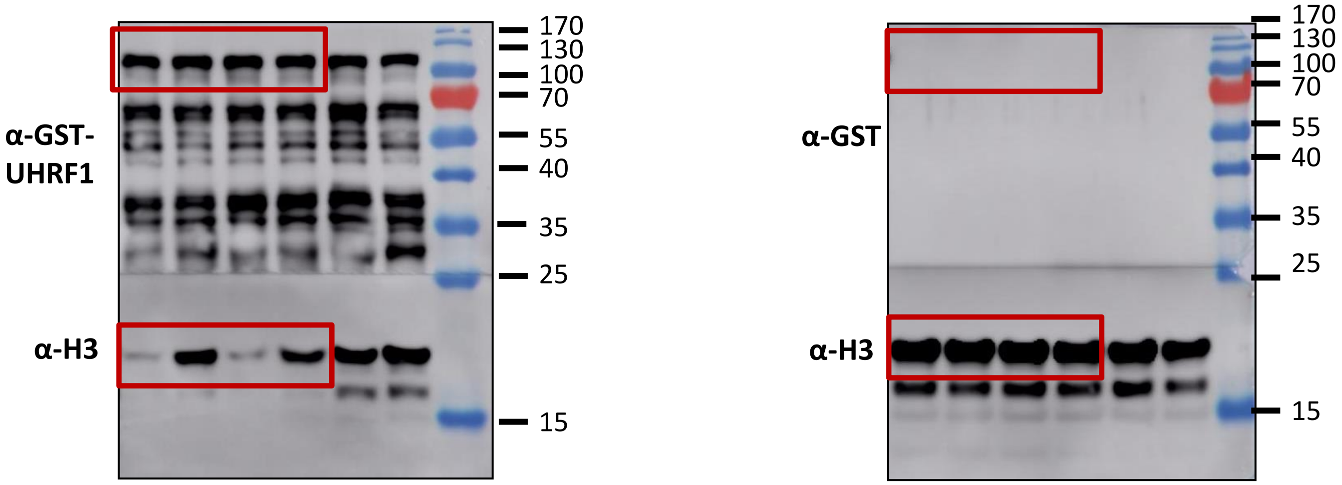

Supplementary Figure 5. Whole uncropped Western blots for Figure 5. Molecular weight markers are as indicated. Labeling and order are consistent with Figure 5.
